# Randomised, sham-controlled, double-blinded, multicentre international trial to evaluate the efficacy of the Ventfree Respiratory Muscle Stimulator to assist ventilator weaning in critically ill patients: a study protocol of a randomised controlled trial

**DOI:** 10.1136/bmjopen-2025-113540

**Published:** 2026-04-21

**Authors:** Euan J McCaughey, Angus J McLachlan, Jason Cai, Guillermo Cohen Freue, Alexandre Demoule, Deepa B Gotur, Nicholas S Hill, Christopher Dimatteo, Senen Pena Oliva, Mayur B Patel, Timothy D Girard, Leo Heunks

**Affiliations:** 1 Liberate Medical LLC, Crestwood, Kentucky, USA; 2 National Spinal Injuries Unit, Queen Elizabeth University Hospital, Glasgow, UK; 3 University of Glasgow, Glasgow, Scotland, UK; 4 Octech Medical, St Paul, Minnesota, USA; 5 La Pitié-Salpêtrière Université Hospital, Sorbonne Université, Paris, Île-de-France, France; 6 Houston Methodist Hospital, Houston, Texas, USA; 7 Tufts Medical Center, Boston, Massachusetts, USA; 8 Critical Illness, Brain dysfunction, and Survivorship (CIBS) Center, Vanderbilt University Medical Center, Nashville, Tennessee, USA; 9 University of Pittsburgh School of Medicine, Pittsburgh, Pennsylvania, USA; 10 Radboud University Medical Center, Nijmegen, The Netherlands

**Keywords:** Adult intensive & critical care, Electric Stimulation Therapy, Ventilators, Mechanical, Respiratory Function Test, Intensive Care Units

## Abstract

**Introduction:**

Nearly half of patients who receive invasive mechanical ventilation for acute respiratory failure require over 4 days of ventilator support, each day of which is associated with increased morbidity, mortality and cost. Many of these patients develop expiratory muscle atrophy and weakness, which are linked to failed extubation and weaning. We seek to test the hypothesis that exhalation synchronised abdominal functional electrical stimulation reduces mechanical ventilation duration.

**Methods and analysis:**

This pivotal superiority trial will be performed in up to 30 intensive care units (ICUs) in the USA, France, the Netherlands and Australia. Adults (≥22 years old) who have been mechanically ventilated for 24–96 hours and are expected to remain ventilated for another 24+ hours are potentially eligible. We will recruit participants until 150 successful liberations from mechanical ventilation occur. To achieve this, we estimate that a maximum of 272 participants will be randomised in a 1:1 ratio to receive 30 min of active exhalation synchronised abdominal functional electrical stimulation (vs sham). The intervention will be applied using the VentFree Respiratory Muscle Stimulator two times per day, a minimum of 5 days per week, for a maximum of 28 days or until ICU discharge. The primary outcome is time from first intervention to successful liberation from mechanical ventilation. Secondary outcomes include cough peak flow (CPF) and maximum expiratory pressure (MEP) at 24 hours post-extubation, hospital and ICU length of stay, reintubations, complications, ICU readmissions, 90-day mortality and quality of life. The participant, clinical team and outcome assessor are blinded to group allocation. A positive outcome has the potential to improve patient-centred outcomes in ICUs.

**Ethics and dissemination:**

This study was approved by local ethics institutions in the USA, Australia, France and the Netherlands. We describe the methods herein using the Standard Protocol Items for Randomised Trials framework and discuss key design decisions. The results will be disseminated through peer-reviewed journal publications, conference presentations and clinicaltrials.gov updates. Individual country-level approvals are as follows:

France:

- Ethics committee: Comité de Protection des Personnes Ile-de-France X.
- Reference numbers: CPP 27-2024; RCB 2024-A00559-38.
- Initial approval date: 14 May 2024.

Australia:

- Ethics committee: South Eastern Sydney Local Health District Human Research Ethics Committee.
- Reference number: 2022/ETH02724.
- Initial approval date: 21 March 2023.

Netherlands:

- Ethics committee: Medisch Ethische Toetsings Commissie Erasmus MC.
- Reference numbers: MEC-2023-0364; NL84195.000.23.
- Initial approval date: 30 April 2024.

USA:

- Ethics committee: WCG IRB.
- IRB tracking number: 20214073.
- Initial approval date: 13 March 2023.

All participating sites are currently approved and operating under protocol version 09 or later.

**Trial registration number:**

NCT05759013. Registered 8 March 2023.

**STRENGTHS AND LIMITATIONS OF THIS STUDY**

- The standard weaning protocol developed for this trial will improve consistency across the 30 study sites.
- The screening method for awakening and comprehension will improve the quality of pulmonary function data collected in the trial.
- The international design of the trial, which includes three continents, will improve generalisability of the trial results.
- The optimum stimulation parameters for abdominal functional electrical stimulation have yet to be established and, as such, the treatment protocol has been chosen based on physiological and practical considerations.

## Introduction

 Mechanical ventilation is a life-saving intervention for patients with acute respiratory failure,^1^ but this invasive medical therapy has been linked to adverse and often life-threatening complications including ventilator-induced lung injury, muscle weakness and delirium.^2^,^5^ The probability of survival decreases as duration of ventilator support increases^1^ with prolonged intensive care unit (ICU) stays associated with functional disability and increased mortality up to 1 year post discharge.^3^ Accordingly, treatments that reduce duration of mechanical ventilation may also reduce morbidity and mortality, improve quality of life and reduce healthcare costs.

The respiratory muscles play an important role in liberation from mechanical ventilation,^6^,^11^ with expiratory muscle strength an independent predictor of liberation success^12^,^16^ and delayed extubation.^13^ In addition, an effective cough predicts liberation success, morbidity and mortality.^17^,^19^ However, ~40% of mechanically ventilated patients lose >10% of diaphragm mass^8^ and 30% experience a loss of abdominal muscle mass^10^ during the first 5 days of mechanical ventilation. This results in a large reduction in force generation,^5 7 8^ with these patients experiencing considerable expiratory muscle weakness and a reduction in respiratory function.^10 19 20^ Therefore, improvements in expiratory muscle strength have the potential to reduce duration of mechanical ventilation and increase the likelihood of successful ventilator liberation.

Inspiratory muscle training using a threshold loading device^21^ and temporary transvenous phrenic nerve stimulation^22^ are two therapies that target respiratory muscle atrophy during mechanically ventilated patients. While the benefit of inspiratory muscle training for critically ill patients remains uncertain,^23 24^ this therapy depends on patient motivation and cooperation, which can limit its effectiveness during critical illness. Temporary transvenous phrenic nerve stimulation has been shown to improve maximal inspiratory pressure, but this finding has not yet translated to an increased proportion of patients being liberated from mechanical ventilation.^22^ Furthermore, this invasive technique requires a central venous catheter fitted with pacing electrodes.

Neuromuscular electrical stimulation, the application of small electrical pulses to the motor nerves supplying a muscle to elicit a contraction, has been shown to be a safe and effective technique to reduce muscle atrophy and critical illness polyneuromyopathy in critically ill patients.^25^ When neuromuscular electrical stimulation is applied to the abdominal wall muscles in synchrony with exhalation, termed abdominal functional electrical stimulation, the effect on ventilation is similar to a physiological contraction of the abdominal wall muscles. Previous studies have shown that abdominal functional electrical stimulation can acutely improve respiratory function^26^ and increase tidal volume and reduce duration of mechanical ventilation for people with tetraplegia.^27^ Importantly, abdominal functional electrical stimulation does not require patient cooperation.

We completed two pilot clinical trials that examined abdominal functional electrical stimulation using the VentFree device during invasive mechanical ventilation in critically ill patients.^28 29^ Both studies demonstrated the feasibility of applying this technology with critically ill mechanically ventilated patients. Notably, median ventilation duration and ICU length of stay were 10 versus 52 days (p=0.07) and 12 versus 54 days (p=0.03) for the intervention versus sham group, respectively. No serious device-related adverse events occurred, and treatment session compliance was >90%.^30^

The primary objective of the Pivotal Evaluation of Abdominal Functional Electrical Stimulation using the VentFree device for Weaning from Mechanical Ventilation (PREVENT) trial is to determine whether using the VentFree device reduces the time from first treatment to successful liberation from mechanical ventilation in critically ill adult patients.

## Methods

### Trial design

PREVENT is a randomised, sham-controlled, participant and assessor-blinded, multicentre clinical trial. It uses a superiority design to compare active and sham abdominal functional electrical stimulation on mechanical ventilation duration.

### Study setting

Participants will be enrolled in up to 30 sites in the USA, France, the Netherlands and Australia. No more than 25% (68 participants) of the study population will be enrolled at any single site and no more than 45% of participants outside the USA. A full list of all participating sites can be found at https://clinicaltrials.gov/study/NCT05759013.

### Eligibility criteria

#### Inclusion criteria

Participants must meet both of the following inclusion criteria:

1. ≥22 years of age (as per American medical device regulations).
2. Invasively mechanically ventilated ≥24 hours.

#### Exclusion criteria

Participants must meet none of the following exclusion criteria:

1. Invasively mechanically ventilated for >96 hours prior to randomisation.
2. Expected to be disconnected from mechanical ventilation ≤24 hours after enrolment.
3. Intubated for ≥24 hours during a prior episode of invasive mechanical ventilation during the current hospitalisation.
4. Body mass index (BMI) ≥40 kg/m^2^.
5. No contraction of abdominal wall muscles in response to abdominal functional electrical stimulation (determined by ultrasound).
6. Pre-existing neuromuscular or muscular disorder that could affect the respiratory muscles (eg, spinal cord injury or Guillain-Barré syndrome).
7. Open abdominal surgery <4 weeks prior to enrolment.
8. Open or damaged skin at the area of electrode placements.
9. Pacemaker and/or implanted electronic device.
10. Known or expected pregnancy.
11. Pharmacological paralysis at the time of enrolment (those receiving neuromuscular blockers may be enrolled after a ≥12-hour washout period).
12. Tracheostomy prior to enrolment.
13. On home non-invasive ventilation (except for Continuous Positive Airway Pressure (CPAP) or Bilevel Positive Airway Pressure(BiPAP) for obstructive sleep apnoea).
14. Receiving or expected to receive comfort measures (palliative, hospice, comfort care, etc.) at the time of screening or enrolment.
15. Participation in any of the following:
  - A study with the same or similar primary outcome.
  - A study investigating electrical stimulation or respiratory muscle therapy.
  - Any study which the site principal investigator (PI) determines may interfere with the results of this study.
16. Unable or unwilling to comply with protocol requirements, including assessments, tests and follow-up visits.
17. Any other medical condition which, in the opinion of the investigator, will make participation medically unsafe or will interfere with the study results.
18. Inability to obtain written informed consent from the participant or their legally authorised representative.

### Enrolment and consent

Prior to enrolment, a potential participant, or their legally authorised representative (LAR) for participants who are unable to provide consent due to lack of capacity, will be fully informed of the nature of the trial, details of study procedures, anticipated benefits and potential risks of study participation by the site PI or a suitably trained delegate such as a research co-ordinator. Written informed consent will be provided by the participant/LAR to the site PI or a suitably trained delegate before any study-specific procedures (including screening and baseline tests) are performed. The master participant consent form is provided as online supplemental appendix A. For sites with e-consent capabilities, the signature of the LAR may be obtained electronically. Patients previously incapacitated will be invited to reconsent once they are judged by the site PI to have regained decision-making capacity. No incentives (financial or otherwise) were offered for this trial.

### Eligibility screening

All participants who meet the study inclusion criteria will be pre-screened prior to obtaining informed consent. After a patient’s medical history has been reviewed, informed consent will be obtained from potentially eligible patients before screening is completed. Women of childbearing potential will undergo a pregnancy test. Patients who meet all eligibility criteria will then undergo a test VentFree intervention session to determine if there is contraction of the abdominal wall muscles.

**Figure 1:**
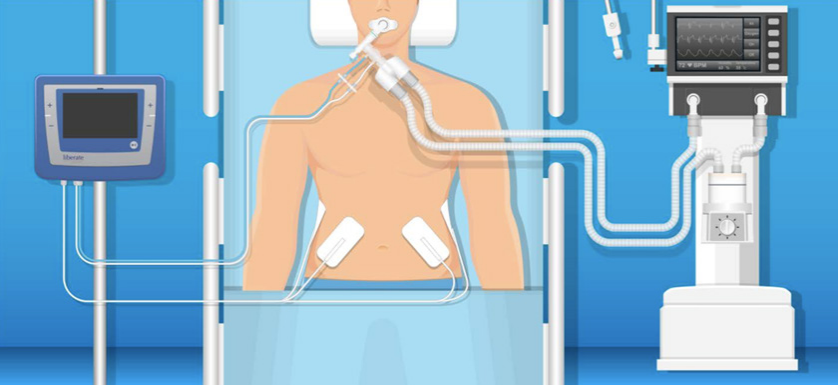
Illustration of the VentFree Respiratory Muscle Stimulator setup. The image shows a participant connected to mechanical ventilation with stimulation triggered using a flow sensor connected to ventilator tubing. Alternatively, stimulation can be triggered using a nasal cannula (not shown).

Electrodes will be placed posterior laterally over the abdominal wall designed to activate the transversus abdominis and internal and external oblique muscles (figure 1).^29^

First, the sham group intensity will be determined during this screening abdominal FES session. This will be conducted by administering the abdominal FES at a frequency of 30 Hz, a pulse width of 350 µs and an amplitude of stimulation insufficient to elicit abdominal wall muscle contraction. The initial stimulation amplitude will be set at 10 mA. In the event that ultrasound confirmation indicates muscle contraction at 10 mA, the amplitude will be systematically reduced in increments of 2 mA, with confirmation sought at each step until muscle contraction is no longer observed. This amplitude will serve as the sham stimulation intensity for all sham treatment sessions.

Next, trains of stimulation pulses, 1–2 s in length with incremental intensities (max. 100 mA), will be given to determine the stimulation threshold for tetanic muscle contraction (‘threshold intensity’). To verify contraction, all three layers of the lateral abdominal muscles, namely the internal and external obliques and the transverse abdominis, must be visible on the ultrasound image. A change in the combined thickness of the three muscle layers should be observed in response to stimulation to confirm eligibility for the trial. Stimulation increments will be made based on the participant’s maximum tolerable level. In communicative participants, discomfort associated with the maximum tolerable level will be recorded on a Visual Analogue Scale with pain ratings from 0 (no pain) to 10 (worst pain). In uncommunicative participants, the maximum tolerable level will be determined as the stimulation intensity that results in a Behavioural Pain Scale^31^ score >4 or a Critical-Care Pain Observation Tool^32^ score >2. If stimulation at 100 mA did not result in a visible contraction of the abdominal wall muscles as verified by ultrasound, the patient is ineligible for the trial. Multiple attempts are permissible prior to randomisation as long as the potential participant continues to meet all eligibility criteria. Once eligibility is confirmed, baseline demographic data will be collected.

### Interventions

Participants will be randomly assigned to receive active stimulation delivered using the VentFree device or sham.

VentFree is composed of a control unit, breathing sensors (ventilator circuit flow sensor and nasal-oral cannula), disposable transcutaneous stimulation electrodes and a power supply. The ventilator circuit flow sensor, which is placed between the ventilator tubing and the participant interface (eg, endotracheal tube), is used for all patients who are endotracheally intubated, have a tracheostomy or are receiving non-invasive ventilation. For those liberated from mechanical ventilation, the nasal-oral cannula serves as a flow sensor—which is placed with the nasal prongs inserted in the participant’s nares and the oral prong suspended over their mouth. The nasal-oral cannula can be used together with either a high-flow or conventional oxygen cannula and can be used with most brands of high flow nasal cannula. Using the breathing sensor, the control unit monitors the breathing pattern of the participant to apply functional electrical stimulation in synchrony with exhalation. Figure 1 illustrates VentFree setup when using the ventilator circuit flow sensor. The stimulation electrodes and ventilator circuit flow sensor are replaced every 5 days of treatment and for every treated participant. The nasal-oral cannula is replaced after every treatment session.

All participants will receive 30 min of the VentFree intervention (or sham) twice per day, for a minimum of 5 days per week, for 28 days or until ICU discharge, whichever comes first.

Discharge from the ICU will be defined as the initial occurrence of any of the following:

- Transfer order to a general or surgical floor team; or
- Transfer to a general or surgical floor; or
- Discharge from the ICU to an outside facility or home.

The first intervention session must be initiated ≤2 hours after randomisation, and a minimum of 4 hours is required between treatment sessions. Training will be provided by the sponsor for all trial procedures.

### Treatment group

In the active treatment group, the VentFree device will be applied with a frequency of 30 Hz and a pulse width of 350 μs. During each stimulation session, the stimulation amplitude will be set to 90% of the participant’s maximum tolerable level, determined using the approach previously described on a daily basis.

While stimulation sessions may occur with participants in the prone position, the optimal positioning for stimulation is supine. If stimulation is to be applied with the patient in the prone position, the patient should be rolled to allow electrodes to be positioned as outlined above. It is important to note that following completion of a prone stimulation session, electrodes must be promptly removed, properly stored and retained for subsequent use, if applicable.

If a participant receives neuromuscular blockade during the trial, all stimulation sessions should continue. In this case, there is no required wash-out period (as required before the initial test VentFree session) between the administration of the neuromuscular blockers and VentFree intervention. However, where practical, the stimulation session after the administration of neuromuscular blockers should be delayed for as long as possible.

### Sham group

The sham group will receive abdominal functional electrical stimulation via the VentFree device exactly as described above but with the sham stimulation amplitude determined at the eligibility screening. This amplitude is specifically designed to not cause an active muscle contraction.

### Criteria for discontinuing or modifying allocated interventions

Criteria for discontinuing or modifying allocated interventions are shown in table 1.

**Table 1:** Criteria for discontinuing or modifying allocated interventions

The active or sham treatment must not be initiated if any of the following conditions are met:	Initiated treatment sessions must be paused if any of the following conditions are met:
Heart rate <50 beats per minute or >130 beats per minute	Heart rate <50 beats per minute or >130 beats per minute
Mean arterial pressure <60 mm Hg or >110 mm Hg (with or w/o vasopressors)	Mean arterial pressure <60 mm Hg or >110 mm Hg (with or w/o vasopressors)
Respiratory rate >35 breaths per minute	Respiratory rate >35 breaths per minute
SpO_2_ <90% (with ventilator support, O_2_ supplementation)	SpO_2_ <90% (with ventilator support, O_2_ supplementation)
BPS >4 or CPOT >2	Participant experiences a seizure
Participant refuses initiation of treatment	Participant requests to stop
	Increase in heart rate >20 beats per minute
	Increase in systolic blood pressure >20 mm Hg

BPS, Behavioural Pain Scale; CPOT, Critical-Care Pain Observation Tool; O2, Oxygen; SpO2, Peripheral Oxygen Saturation.

If a participant experiences a seizure during a treatment session, the session must be ended. If any of the other above-mentioned criteria are met causing the treatment to be paused, the participant should be re-evaluated every 2 min up to a maximum of 10 minutes to determine if their conditions have stabilised to within the stopping criteria parameters. If at any time during the 10-minute re-evaluation period, the participant’s parameters have returned to be within the above-mentioned criteria, the treatment can be resumed.

Participation in the trial is strictly voluntary. A participant, or their LAR, has the right to withdraw from treatment or the study at any time for any reason. The investigator also has the right to terminate the treatment or participation of a participant due to transfer to another institution, significant adverse events, any other reason relating to the participant’s safety or integrity of the trial data, participant non-compliance with the study assessments or procedures, or any problem deemed by the investigator to be sufficient to cause discontinuation from treatment or study participation. The site PI will inform the participant of the withdrawal.

### Strategies to improve adherence to interventions

Stimulation parameters are titrated to avoid participant discomfort at every treatment session. We believe this is an effective way to improve adherence in this trial. Furthermore, after the first week, stimulation is only applied on 5 of 7 days to hopefully improve adherence.

### Relevant concomitant care permitted or prohibited during the trial

All participants will continue to receive standard of care throughout the trial. There will be no limitations placed on the care a participant can receive during the trial. To reduce variability in mechanical ventilation weaning practices, all sites will use a standardised weaning protocol (online supplemental appendix B).

### Provisions for post-trial care

Participants will not receive the intervention beyond day 28 or ICU discharge, whichever comes first. All sites, and the trial sponsor, have appropriate insurance provisions in place to ensure protection of all participants.

### Outcomes

#### Primary outcome

The primary outcome is time (in days) from first administration of the VentFree intervention to successful liberation from mechanical ventilation, defined as disconnection from mechanical ventilation that does not require invasive mechanical ventilation in the subsequent 7 days after disconnection,^33^ or until ICU discharge (as defined above), or until live hospital discharge, whichever occurs first.

#### Secondary outcomes

Key secondary outcomes are CPF (minimum 24 hours post-extubation once the patient meets awakening criteria^13 34^) and MEP (minimum 24 hours post-extubation once the patient meets awakening criteria^13 34^). The best (maximum value) of three attempts will be used in the analyses of each individual parameter.^34^ The full pulmonary function testing protocol is provided in online supplemental appendix C.

Other secondary outcomes are:

1. Incidence of device-related adverse events (hospital discharge).
2. Time from first administration of the VentFree intervention to ICU discharge.
3. Time from first administration of the VentFree intervention to hospital discharge.
4. Incidence of successful liberation from mechanical ventilation (day 28 or ICU discharge, whichever comes first).
5. Incidence of reintubations (ICU discharge, 90 days post treatment).
6. Incidence of readmissions to the ICU (90 days post treatment).
7. Incidence of readmissions to the hospital (90 days post treatment).
8. Incidence of acute respiratory infections (hospital discharge).
9. Incidence of hospital-acquired infections (hospital discharge).
10. Incidence of tracheostomy (ICU discharge).
11. Mortality (hospital discharge, 90 days post treatment).
12. Maximum inspiratory pressure (minimum 24 hours post extubation once the patient meets awakening criteria).
13. Mobility as assessed by the ICU Mobility Scale^35^ (ICU discharge).
14. Quality of life as assessed by the 5-level EQ-5D version (EQ-5D-5L)^36^ (90 days post treatment).

**Figure 2:**
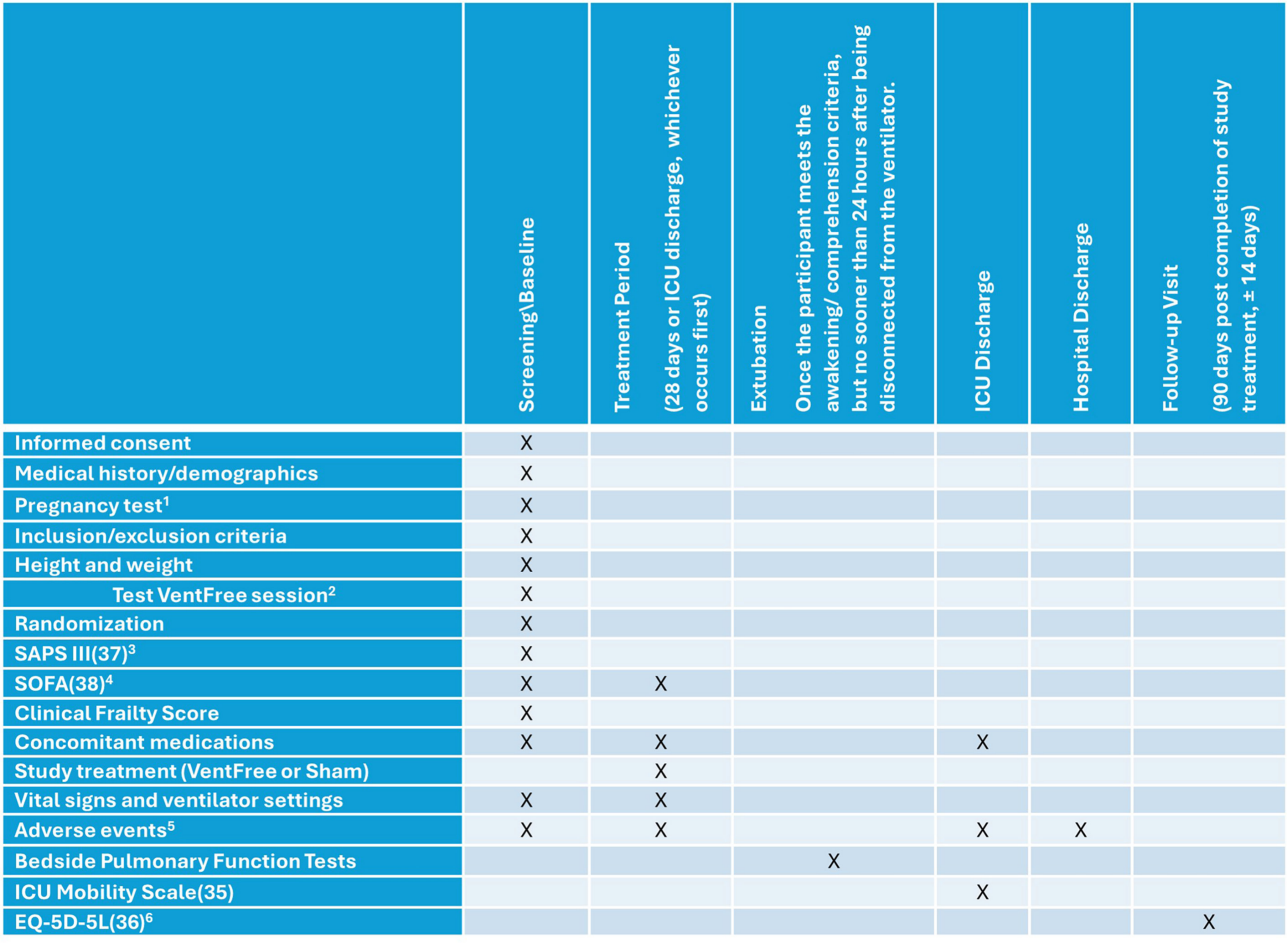
Participant timeline: schedule of enrolment, interventions and assessments. ^1^For women of childbearing potential. ^2^Participants who have no contraction of the abdominal wall muscles in response to abdominal functional electrical stimulation are not eligible for participation. ^3^To be assessed as of ICU admission. ^4^To be assessed as of ICU admission, first day of stimulation and every third day during the treatment period. ^5^Adverse Events (AEs) will be collected from the time of informed consent until hospital discharge. ^6^If any participant’s score is 4 (severe) or 5 (extreme) in any section of the survey (mobility, self-care, usual activities, pain/discomfort), the researcher will notify the PI and instruct the participant to follow up with their primary care physician for further instructions. ICU, intensive care unit; PI, principal investigator. AEs: Adverse Events, EQ-5D-5L: The 5-level EQ-5D version, SAPS III: Simplified Acute Physiology Score (SAPS) 3, SOFA: The Sequential Organ Failure Assessment.

The full schedule of assessments is shown in the participant timeline in figure 2.

### Follow-up visit

A follow-up visit is required 90 days (± 14 days) from the date of the final study treatment. The visit may be conducted in clinic or by phone or videoconference. The visit will record EQ-5D-5L,^36^ new hospitalisations, new intubations and mortality. If any participant’s score is 4 (severe) or 5 (extreme) in any section of the EQ-5D-5L, the research staff will notify the PI and instruct the participant to follow up with their primary care physician for further instructions.

### Sample size

Assuming an outcome expected subdistribution hazard ratio (active/sham) of 1.7, 1:1 group allocation, a type I error rate of 5% (two-sided), and 90% power, the targeted total number of participants successfully liberated from mechanical ventilation is 150. Based on the results of pilot studies, it is estimated that at least 55% of participants will achieve successful liberation. Thus, approximately 272 participants will be needed for the trial. The trial will continue to randomise participants until 150 events from the combined study groups have occurred, regardless of the total number of participants randomised. The final analysis will be performed after all randomised participants have completed or discontinued their participation in the study.

### Recruitment

All consecutive admissions will be screened against the eligibility criteria. If a patient is identified as being potentially eligible for the trial, their treating clinician will alert the study team. The study team will then approach the patient and provide them, or their LAR, with information about the trial as outlined in the Enrolment and consent section. We have estimated a recruitment period of 24 months for this trial. With the expectation of 30 sites, and a target sample size of ~272, this would require recruitment of 0.37 participants per site per month.

### Sequence generation

Participants will be considered enrolled in the study once they or their LAR have signed the informed consent form, passed the screening eligibility test, met all eligibility criteria for the study and are randomised to a treatment assignment. A web-based electronic database capture (EDC) system will be used to randomly assign participants in a 1:1 ratio to active or a sham treatment. Randomisation will be stratified by country, with varying block sizes. Prior to trial initiation, an independent statistician who is not involved in the clinical aspects of the trial will generate the randomisation schedule. Randomisation will be requested by a suitably qualified local investigator for each participant. Randomisation sequences will be concealed within the EDC, with only an independent statistician having access to the allocation schedule.

### Blinding

Participants, individuals involved in clinical decision-making and outcome assessors will be blinded to treatment allocation and will remain blinded until the database is locked. Unblinded clinicians responsible for administering active or sham treatments will draw each participant’s bedside curtain while preparing the active device. The VentFree device dims the displayed value of the stimulation current stimulation 15 s after the last current adjustment made, and the participant’s abdomen will be covered with a bed sheet so that family members, caregivers and outcome assessors are less likely to determine treatment assignments. Additionally, participants and their LAR will receive instructions not to discuss their perceptions or sensations of the treatment with others, including other participants, outcome assessors and individuals involved in clinical decision-making. This information will be reiterated a number of times during the intervention period to account for the fact patients may have been sedated/delirious at the time of enrolment.

In a case where unblinding is required, the local investigator should contact the sponsor, who can provide the site with information relating to a participant’s allocation upon reasonable request.

### Data management

Clinical data will be collected in web-based standardised electronic case report forms (eCRFs). The Food and Drug Administration (FDA) 21 CFR Part 11 and ISO14155:2020 will be followed as well as other applicable legislation on the handling of electronic data. Participant personal information will be pseudonymised. Site and participant numbers will be used to track participant information. Investigators and designated staff will receive training on eCRF completion and use of the EDC system (Viedoc, Uppsala, Sweden) prior to use.

All participant information will be kept confidential according to applicable laws and regulations. Information recorded on paper will be kept in secured locations, and electronic information will be stored on password-protected computers. The electronic data stored for this study will be kept in a database in compliance with 21 CFR Part 11, General Data Protection Regulation and the Health Insurance Portability and Accountability Act. Participant data will not contain details of participant identity. The data will be stored on a secure server and backed up routinely. Study data will be coded to prevent participant identification, except by the institution, investigators and other healthcare personnel involved in the study.

### Statistical methods

#### Competing events

Participants who die, are put on palliative care or are transferred out of the ICU for any reason due to a worsening condition before achieving successful liberation by day 28 or ICU discharge (whichever occurs first) will be considered to have experienced a competing event. The time from FES treatment administration to death, initiation of palliative care or ICU transfer due to worsening condition will be used for analysis. Participants who reach day 28, are discharged from the ICU or withdraw from the study before experiencing successful liberation from mechanical ventilation or a competing event will be considered right-censored at the earliest of day 28, ICU discharge or study withdrawal.

#### Primary outcome

Cumulative incidence competing risk (CICR) estimates will be used to describe rates of successful liberation over time in the presence of a competing event. CICR curves from different treatment groups will be compared by Gray’s test at a two-sided significance level of 0.05.^37^

In the rare event that successful liberation occurs before the first VentFree treatment and any competing events, the participant will be considered as having experienced the event of interest on day 1. If any competing event occurs before both the first VentFree treatment and the event of interest, the participant will be considered as having experienced the competing event on day 1. If a participant drops out of the trial before receiving the first VentFree treatment for any reason other than successful liberation or competing events, the participant will be treated as censored on day 1.

As a supplement to the primary analysis, the cause-specific hazard ratio (*csHR*) and subdistribution hazard ratio (*subHR*) for successful liberation, accompanied by their corresponding CIs, will be computed using Cox proportional hazard model and Fine-Gray subdistribution hazard model, respectively. Robust (sandwich) variance estimators clustered by site will be used in these regression-based models to account for potential within-site correlation in this multicentre trial.

The primary analysis of the primary outcome will be based on the intent-to-treat (ITT) population, which will include all randomised participants, regardless of whether or not abdominal FES is received. Sensitivity analyses will be performed using the modified ITT population, which will include participants who receive at least one VentFree intervention session, and the per protocol population, which will include participants who receive treatment and have no major protocol deviations. In the case of any discrepancies in the conclusions derived from analyses based on different analysis populations, additional analyses will be performed to elucidate these significant differences. No interim analysis is planned.

For supportive purposes, time from the start of invasive mechanical ventilation to successful liberation during the treatment period of 28 days, or ICU discharge, whichever comes first, will be analysed in the same manner as the primary outcome on the ITT population. In addition, an analysis using an alternative definition of successful liberation will be conducted, defined as disconnection from mechanical ventilation without the need for invasive mechanical ventilation within 48 hours following disconnection, or until ICU discharge, whichever occurs first. All statistical methods described for the primary analysis, including CICR estimation, Gray’s test, and the Fine-Gray subdistribution hazards model and cause-specific Cox proportional hazards model, will be applied in the same manner using the same analysis populations.

Treatment effect heterogeneity across sites and geographic regions will be assessed using Fine-Gray subdistribution hazards models including treatment-by-site and treatment-by-region interaction terms. To ensure model stability, sites enrolling fewer than 10 participants will be pooled with other small sites within the same country for the site-level analysis.

Exploratory subgroup analyses based on selected baseline demographic and clinical characteristics will also be conducted. Interaction terms will be evaluated using Fine–Gray models, and subgroup-specific estimates will be reported descriptively. Given that some subgroups may include limited numbers of participants or events, results will be interpreted cautiously and considered hypothesis-generating rather than confirmatory.

#### Key secondary outcomes

The key secondary outcomes—CPF and MEP measured when the participant meets the awakening/comprehension criteria but no sooner than 24 hours after the patient has been extubated—outcome will be sequentially compared between treatment groups using the ITT population in a superiority manner, provided that the active group demonstrates superiority over the sham group in the primary outcome. Each comparison will be conducted at a one-sided significance level of 0.05 using an unpaired t-test. The Wilcoxon rank-sum test will be performed as a supportive analysis to account for any potential violations of the normality assumption.

To control the family-wise type I error, the testing procedure will be carried out in a fixed sequence, starting with the primary outcome, followed by CPF and MEP. The testing will stop at the first rejection failure. This fixed sequence testing procedure guarantees that the family-wise error rate is appropriately controlled across both the primary and key secondary outcomes. Missing data for the key secondary endpoints will be imputed. For participants who have experienced competing events, the worst observed value for that parameter during the study will be imputed. For other participants, under the assumption of missing at random, a linear regression model (multiple imputation) will be used to impute the missing values for CPF and MEP. Predictors in the imputation model will include the following baseline factors: region, race, ethnicity, age, gender, BMI, COPD (yes/no), sepsis (yes/no), clinical frailty scale, steroid use (yes/no) and the Sequential Organ Failure Assessment (SOFA) score. Predictors may be removed if convergence issues arise.

### Oversight and monitoring

The sponsor, Liberate Medical LLC, is acting as the coordinating centre for this trial. All authors are the sole members of the study scientific advisory board. As this study is deemed to be non-significant risk, there is no requirement for a formal data monitoring committee. Instead, an independent physician will serve as the study medical monitor and review all reportable events to monitor for incidence of serious events that would warrant modification or termination of the trial. The medical monitor may recommend study modification or termination because of concerns over participant safety or issues. This notification will be submitted in writing to the Sponsor for consideration and final decision. All protocol changes will be communicated by the sponsor to relevant parties.

### Adverse event reporting and harms

The investigator or designee will record all adverse device events, serious adverse events, serious adverse device events and unexplained adverse device events and device deficiencies in the source document as well as in each participant’s eCRF with information about:

- Diagnosis and relevant clinical findings.
- Date of onset.
- Date of recovery or the current status of adverse event.
- Severity.
- Treatment.
- Relation to investigational product.
- Relation to the investigational procedure.
- Action taken with study treatment.
- Seriousness of the adverse event.

All adverse device events, serious adverse events, serious adverse device events and unexplained adverse device events and device deficiencies will be reported to the sponsor within one business day of being made aware of the event and will be reported to the investigational review board/ethics committee as per applicable regulations.

### Frequency and plans for auditing trial conduct

An independent physician will serve as the study medical monitor and review all reportable events for serious events that warrant modification or termination of the trial. The medical monitor may recommend study modification or termination because of concerns over participant safety or issues. Such a recommendation would be submitted in writing to the Sponsor for consideration and final decision.

Risk-based monitoring activities will be conducted according to the CIP, ISO 14155:2020 GCP, 21 CFR 812.46 and regulatory guidance relevant to this clinical study. Periodic monitoring will be completed on-site and/or by remote visit from representatives of the sponsor or CRO who will check the eCRFs for completeness and accuracy, verify eCRFs with source documents, review site compliance, review administrative records and confirm that all adverse events have been reported as required. In addition, frequent communications by the study monitors will ensure that the investigation is conducted according to the CIP, regulatory requirements and good clinical practice.

Study close-out will be performed by the study monitor upon completion of the study. The close-out visit will consist of reconciliation of all remaining data inconsistencies, obtaining current status determination for all unresolved adverse events and review of administrative records. In addition, the monitor will perform final investigational product reconciliation for all products received, used, shipped or disposed of during the study.

### Dissemination plans

The first publication of the results of this study will be of the pooled study data from all study sites analysed according to the statistical analysis plan. These results will be disseminated through peer-reviewed journal publications, conference presentations and clinicaltrials.gov updates. Additionally, these results will be communicated via press releases and social media posts. De-identified participant-level data will be available upon reasonable request to the study sponsor. The lead PIs TDG and LH, who are world leaders in ICU research, will actively inform national and international patient organisations about the study results through conference discussions and workshops. The study sponsor, Liberate Medical, will also draft a lay summary of the trial that can be provided to study sites and will also be published on their website.

Subsequent to the first publication site, PIs will be encouraged to suggest secondary analyses of the pooled study data. All study PIs will be invited to contribute to suggested pooled secondary analyses suggested by individual site PIs. Results will be disseminated as described above. Sites are also free to use, publish and present data arising from its participation in the study protocol after the primary analysis has been published.

## Discussion

This randomised controlled trial is a pivotal evaluation of the efficacy of abdominal functional electrical stimulation, delivered via the VentFree device, to assist ventilator weaning in critically ill adults. While the design posed a number of challenges, it also offered the opportunity to create innovative solutions to existing problems.

After the cause of respiratory failure has improved and when patients are clinically stable, the current standard of care is to evaluate a patient’s readiness for liberation using a spontaneous breathing trial (SBT), which is typically a period of minimal ventilator support. If the patient does well during the SBT, he/she can be considered for extubation; otherwise, the patient is returned to full mechanical ventilation with the process repeated at a later date.^38^ As such, we have developed a standardised weaning protocol (online supplemental appendix B) that should reduce variability in this study.

The optimum stimulation parameters for abdominal functional electrical stimulation have yet to be established. As such, the treatment protocol has been chosen based on physiological and practical considerations. First, respiratory muscle atrophy has been shown to occur most rapidly within the first week of mechanical ventilation.^7 8^ Therefore, we wish to start the intervention as early as possible within this period. Second, the force evoked from a muscle by electrical stimulation can decline over time because of repetitive activation of the same motor units. Therefore, we reason that limiting the stimulation session duration to 30 min will help to ensure a strong muscle contraction throughout the session. The practical considerations include the availability of research staff on weekends and possible interference with clinical protocols. The exact stimulation frequency, pulse width and amplitudes applied here have been used in a range of other abdominal functional electrical stimulation studies, including our pilot studies.^27 28 30^

As this trial is recruiting participants from up to 30 different ICUs across four different countries, we believe the results will be generalisable globally. Furthermore, we envision that our inclusion and exclusion criteria will enable inclusion of most patients who are at risk of respiratory muscle atrophy due to mechanical ventilation.

### Trial status

Recruitment for the trial was completed in January 2026. Analysis is estimated to begin in April 2026. The first participant was recruited on 15 February 2024. The current protocol is version 09 dated 9 October 2024.

## Supplementary material

10.1136/bmjopen-2025-113540 online supplemental file 1

10.1136/bmjopen-2025-113540 online supplemental file 2

10.1136/bmjopen-2025-113540 online supplemental file 3
