# Supplementary material for: Randomised, sham-controlled, double-blinded, multicentre international trial to evaluate the efficacy of the Ventfree Respiratory Muscle Stimulator to assist ventilator weaning in critically ill patients: a study protocol of a randomised controlled trial
Source: BMJ Open. 2026 Apr 21;16(4):e113540. doi: 10.1136/bmjopen-2025-113540 (PMC13110553; doi:10.1136/bmjopen-2025-113540)
Supplement: online supplemental file 1 [file bmjopen-16-4-s001.pdf]

Protocol # LM-VF-P3
IRB Approved Template
MUST BE APPROVED
FOR SITES BEFORE USE
AS MODIFIED
Oct 22, 2024

INFORMED CONSENT FORM

TITLE: A Randomized, Sham Controlled, Double-blinded, Multi-center Trial to Evaluate the Efficacy of the VentFree Respiratory Muscle Stimulator to Assist Ventilator Weaning in Critically Ill Patients

PROTOCOL NO.: LM-VF-P3
WCG IRB Protocol #20214073

SPONSOR: Liberate Medical, LLC

INVESTIGATOR: Name
Address
City, State Zip
Country

STUDY-RELATED PHONE NUMBER(S): Phone Number
Phone Number (24 hours)
[24 hour number is required]

In this consent form "you" generally refers to the research participant. If you are being asked as the legally authorized representative to permit the participant to participate in the research, "you" in the rest of this form generally means the research participant.

Taking part in this research is voluntary. You may decide not to participate, or you may leave the study at any time. Your decision will not result in any penalty or loss of benefits to which you are otherwise entitled.

If you have any questions, concerns, or complaints or think this research has hurt you, talk to the research team at the phone number(s) listed in this document.

RESEARCH CONSENT SUMMARY

You are being asked for your consent to take part in a research study. This document provides a concise summary of this research. It describes the key information that we believe most people need to decide whether to take part in this research. Later sections of this document will provide all relevant details.

What should I know about this research?

- Someone will explain this research to you.
- Taking part in this research is voluntary. Whether you take part is up to you.
- If you don't take part, it won't be held against you.

- You can take part now and later drop out, and it won't be held against you
- If you don't understand, ask questions.
- Ask all the questions you want before you decide.

How long will I be in this research?

We expect that your taking part in this research will last 3-4 months.

Why is this research being done?

Breathing machines, also known as mechanical ventilators, are used in hospitals to help people with serious conditions to breathe. These machines can save lives, but they can also cause problems. These problems can include lung injury due to the pressure of the machine, lung infections, muscle weakness, and mental health issues. Also, wasting of the respiratory muscles is a significant concern for patients on mechanical ventilation. Studies suggest that this wasting of the respiratory muscles can start as soon as the first day the person is on the ventilator, with most wasting occurring within the first five days of intubation. Respiratory muscle wasting can make it more difficult to get patients off the ventilator, as their muscles may not be strong enough to handle the energy needed to breathe independently.

Being on a ventilator for a long time can have several risks. These include getting lung infections, feeling anxious or developing Post-Traumatic Stress Disorder (PTSD), damage to the airways from the breathing tube, becoming weak in both the breathing and other muscles, and possibly becoming dependent on the ventilator to breathe.

Also, when someone depends on the ventilator, it's harder for them to do rehab exercises and it can delay their discharge from the ICU. Staying too long in the ICU can also lead to worse health and higher chances of dying within a year after leaving the hospital.

Neuromuscular electrical stimulation applied to the abdominal wall (called abdominal NMES) is the application of electrical pulses to the abdominal muscles. The abdominal muscles are one of the main groups of muscles that help you breathe out. When NMES is applied to your abdominal muscles while you breathe out, it is referred to as Functional Electrical Stimulation (FES). Functional Electrical Stimulation is a technology that has been used on many tens of thousands of people since the 1960's to help with stroke, pain and spinal cord injury. Functional Electrical Stimulation can strengthen the breathing muscles and improve breathing. Over 1,560 patients have received abdominal FES in various studies, across a broad range of respiratory conditions, including for people who require mechanical ventilation. It has been shown to be a well-tolerated therapy with minimal side effects. We anticipate that less than 5% of patients will experience any form of side effects, very few of which will require any medical intervention.

The purpose of this study is to explore whether an investigational abdominal FES device, when applied with exhalation (breathing out), is safe and effective in reducing the amount of time you have to spend on the ventilator.

What happens to me if I agree to take part in this research?
If you decide to take part in this research study, the general procedures include randomization to the investigational procedure of FES or a sham procedure that does not use FES. You will have the procedure done for 30 minutes, twice per day, for a minimum of five days per week, for 28 days, or until you are discharged from the ICU (whichever comes first). Vital signs and medical information will be collected from you, and you will have tests to see how well you can breathe.

Could being in this research hurt me?
The most important risks or discomforts that you may expect from taking part in this research include discomfort, skin irritation and/or burns, increased blood pressure, respiratory rate and/or heart rate, difficulty breathing and electrical shock.

Will being in this research benefit me?
The most important benefits that you may expect from taking part in this research include removal from the ventilator sooner.

Possible benefits to others include improved knowledge for future treatment of patients who need mechanical ventilation.

What other choices do I have besides taking part in this research?
Instead of being in this research, your choices may include weaning without the use of FES.

DETAILED RESEARCH CONSENT

WHY AM I BEING ASKED TO READ THIS FORM?

You are being invited to take part in a research study. This consent form contains important information to help you decide whether to participate in the study.

Taking part in this study is entirely voluntary. If you join the study, you can decide to stop your participation at any time. No one can promise that a study will help you.

After reading and discussing the information in this consent you should know:
- Why this research is being done
- What will happen during the study
- The possible risks and benefits
- What you will be responsible for
- Other options you could choose instead of being in this study
- How your personal health information will be treated during and after the study
- Whether being in this study could involve any cost to you
- What to do if you have problems or questions about the study

Please read this consent form carefully and discuss any questions about this study with the study staff. Talk to your family and friends about it and take your time to make your decision. If you decide to participate, you must sign this form to show that you want to take part.

WHAT IS THE PURPOSE OF THIS RESEARCH?

Neuromuscular electrical stimulation applied to the abdominal wall (called abdominal NMES) is the application of electrical pulses to the abdominal muscles. When NMES is applied to your abdominal muscles, it is referred to as Functional Electrical Stimulation (FES). Functional Electrical Stimulation can strengthen the breathing muscles and improve breathing. The purpose of this study is to explore whether abdominal FES, when applied with exhalation (breathing out), is effective in gradually removing you from the ventilator (referred to as weaning).

You are being invited to take part in this research study because you require mechanical ventilation as a result of a critical illness.

HOW MANY PARTICIPANTS WILL BE IN THE STUDY?

Approximately 272 people will take part in this research at up to 30 different hospitals in the United States, Netherlands, France, and Australia.

WHO IS PAYING FOR THIS STUDY?

This study is funded by the US Department of Defense (DoD). This study is being sponsored by Liberate Medical, a medical device company in the United States (Crestwood, Kentucky). This is the company that makes the VentFree™ Respiratory Muscle Stimulator, which is the device used to provide abdominal FES in synchrony with exhalation in this study. The sponsor is providing money to this hospital to carry out the research study, but no money is paid directly to study doctors or other staff.

WHAT IS THE STUDY DEVICE?

The FDA has determined that this clinical study qualifies as a non-significant risk device study which means that the FDA thinks this technology is very unlikely to cause serious harm to any patients taking part in this study. However, the VentFree is an investigational device which means it is being studied and neither cleared nor approved by the US Food and Drug Administration (FDA). Data is being collected on the VentFree for submission to the FDA.

The VentFree is a non-invasive device that has been under clinical development since 2015 undergoing several studies to test its safety and effectiveness. During these studies, which included healthy volunteer patients and patients on mechanical ventilation, no serious side

effects were recorded, affirming the device's safety in a controlled setting. These studies have also suggested that the device can improve breathing function and reduce the length of time that people spend on mechanical ventilation. Additionally, approximately 50 patients on mechanical ventilation and 50-100 non ventilated patients have been treated with this device in Europe and the USA without any reported serious side effects.

The VentFree device exercises the abdominal muscles, one of the major muscles used for breathing. It applies abdominal FES as the ventilator breathes out air from your body. This is done by placing electrodes (painless sticky pads) on your stomach, which deliver small electrical pulses (like a TENS unit) to your nerve endings that supply the abdominal muscles. The aim is to keep your breathing muscles as strong as possible.

VentFree is made up of a control unit, electrodes, flow sensor, and a nasal-oral cannula (tube). The flow sensor is placed between your endotracheal tube (a flexible plastic tube that is placed through the mouth into the trachea, or windpipe) and the y-piece of the ventilator. Using the flow sensor, the control unit monitors your breathing pattern to apply FES as you breath out.

Because a flow sensor can't detect breathing efforts after you are taken off the ventilator, a nasal/oral cannula will be used for FES treatment until you leave the ICU. A nasal/oral cannula has parts that go near your mouth and nose similar to a tube used to provide oxygen, but no oxygen is provided through this cannula. These cannulas can sense breathing by detecting pressure changes when the patient breathes in and out. These signals are similar to the ones from the flow sensor and are sent to the VentFree device.

The picture below shows how the VentFree is set up.

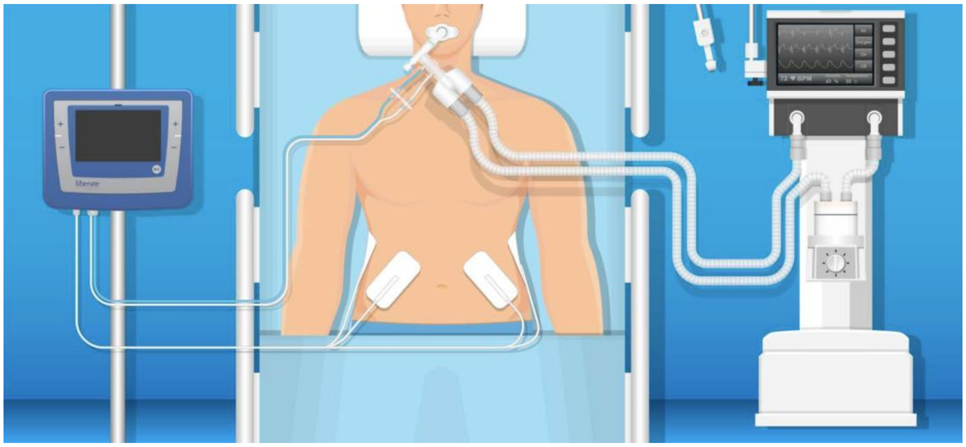

HOW LONG WILL I BE IN THE STUDY?

If you agree to take part in this study, you will be in the study for about 3-4 months. Your participation will last while you are in the hospital and for 3 months after you have completed your study treatment. At the end of the study period, you will complete a final study visit, which can be completed in person or by phone.

WHAT HAPPENS DURING THE STUDY?

This is a randomized, sham controlled, double-blinded study. A randomized study is when study participants are put into groups and given different treatments, and the results are compared to see whether one treatment is better. To ensure the groups are similar, a computer randomly assigns participants into one of the study groups, like flipping a coin to decide which group you should be in. This means that neither you nor the study doctor can decide which treatment you will receive. This study is randomized 1:1, which means you have an equal chance of being put into either group.

In this study you will be randomized to receive abdominal FES treatment or a sham treatment. A sham treatment looks like the real treatment, but no actual treatment is given. The VentFree will be used for both abdominal FES and sham treatment. If you are randomized to sham treatment, the clinician goes through the motions of giving abdominal FES treatment with the VentFree™ device without actually performing it so that you can't tell the difference. It will appear and feel like real abdominal FES.

A double-blinded study is when neither the study doctor nor participants know which treatment the participant is receiving. However, if the study doctor needs to find out what treatment you are receiving to give you proper care, he/she can do so.

If you agree to participate in this trial and sign this form you will be asked to undergo the following procedures:

- You will be asked questions about your medical history to determine if you qualify for the study. This means that you may not be able to participate even if you want to.
- If you are a woman who is able to have children, you will be given a pregnancy test. You will not be able to participate if you are pregnant.
- If you meet all other qualifications, you will have a “test” abdominal FES session to see if the VentFree™ contracts your abdominal muscles and to find the level of stimulation needed to do so. Ultrasound will be used during the test session to see your muscles as they are being stimulated. The ultrasound is given by applying gel to your skin and then placing the ultrasound transducer on your skin to view the muscles inside. You will not be enrolled in study if the test shows no contraction of your muscles or if you have discomfort.
- Once it is determined that you qualify to take part in the study, demographic and baseline information will be collected, such as a list of the medications you are taking

and your height and weight.

- You will be randomly assigned to abdominal FES treatment or sham treatment. You will receive abdominal FES or sham treatment for 30 minutes, twice per day, for a minimum of five days per week, for 28 days or until you are discharged from the ICU, whichever comes first. The study staff person who administers your treatment will know what you have been assigned to so that they can give you the right treatment, but you, the study doctor, and other study staff will not know your treatment assignment. To help maintain the blind, you should not discuss your opinion about your treatment assignment with study staff or other ICU staff.
- Vital signs will be measured during each treatment session, including blood pressure, heart rate, respiratory rate, and pulse oximetry, which is a noninvasive and painless test done with a clip-like sensor most commonly put on the end of a finger, to check your oxygen levels.
- The study staff will collect information about you and your treatment throughout your stay in the hospital, including what medications you are taking and about your daily dietary and fluid intake. It is very important to tell the study staff how you are feeling and if you have any problems or discomfort during or after your treatment sessions.
- You will complete bedside pulmonary function tests to see how well your lungs are working. This involves breathing into a mouthpiece connected to a device that will provide measurements about how you are breathing. A clinician will give you instructions on how to perform the tests (e.g., take a deep breath and cough as hard as possible). These tests will be completed approximately 24 hours after you are removed from the ventilator. If you have to go back on the ventilator, you will be asked to repeat these tests after being removed the second time.
- You will complete a final study visit about 3 months after you have completed the study treatment with VentFree™. This can be completed in person or by phone. During the visit the study staff will ask you questions about your health, any medication changes you may have had, and if you have made any additional trips to the hospital. Just like in the hospital, it is important for you to tell the study staff about your health and if you have had any problems since leaving the hospital. During the visit you will also complete a questionnaire about your health and your quality of life.

You should contact your study doctor or the research staff anytime between your discharge from the hospital and your final study visit to report any health problems. It is very important that you tell the study staff about anything that has changed, so that it can be properly recorded and you can be treated appropriately. Please make sure you tell study staff as much information as possible.

Treatment with the VentFree™ device is experimental. The ultrasound and bedside pulmonary function tests are not experimental but are only being completed for study purposes. All other tests and procedures in this study are not experimental and are considered standard treatment for patients who are mechanically ventilated.

Protocol # LM-VF-P3
IRB Approved Template
MUST BE APPROVED
FOR SITES BEFORE USE
AS MODIFIED
Oct 22, 2024

WHAT ARE MY RESPONSIBILITIES?

For this study to be successful, it is important that you cooperate fully with the study doctor and staff and follow their instructions precisely. If you decide to participate, you need to:

- Answer questions about your medical history and current health status.
- Cooperate with the study procedures as described above.
- Attend the final study visit (in person or by phone) within the timeframe communicated to you by the study staff and complete the questionnaire.
- Inform your study doctor about any health problems, accident or medical intervention that happens while you are in the study, even after you leave the hospital. Changes to your health must be reported even if you think it is not important.
- You need to inform your study doctor if you decide not to continue in the study. You don't have to give a reason for your decision.

WHAT ARE THE POSSIBLE RISKS OR DISCOMFORTS OF BEING IN THE STUDY?

All medical procedures involve some risk of injury. In spite of all reasonable precautions, you might develop side effects from the abdominal FES or sham treatment or from being in the study. These may vary from person to person. You can be treated for any side effects that you have to make them less bothersome or go away. Many side effects go away after treatment with the VentFree™ is stopped, but in some cases, the side effects may be serious and/or lasting. You must inform the study doctor immediately if you experience any negative effects, complications, or injuries while taking part in the study.

****ALL SITES:** The following risk information from [START] through [END] cannot be altered without submission of supporting documentation and/or Sponsor approval of changes. Submitted changes without appropriate documentation will be reverted during Board review.

[START] VentFree™ Side Effects (applies to abdominal FES and sham treatment)

Occasional, remote and improbable side effects are described below. The risk of each side effect is provided, based on use of other muscle stimulators and data collected in previous human studies of VentFree™.

The following side effects are defined as OCCASIONAL (may occur at irregular or frequent intervals within lifetime of device):

Occasional Side Effect
Patient distress from overly strong muscle contraction caused by stimulation, due to<br>a. device error<br>b. use error
Patient distress from excessive stimulation amplitude:<br>a. device error

b. user error
Increased blood pressure due to unwanted stimulation of the celiac plexus
Abdominal Muscle Fatigue or Injury due to excessive muscle contraction
Patient receives inadequate treatment, due to stimulation algorithm not stimulating during exhalation.
Muscle soreness from overuse

The following side effects are defined as REMOTE (rarely occur or occur one time in decade (10 years) or lifetime of device):

Remote Side Effect
Skin Burn
Electric Shock
Patient receives inadequate treatment, due to:<br>a. device error and not delivering treatment<br>b. stimulation not causing a muscle contraction
Patient distress from stimulating during inhalation
Patient distress from erratic timing of stimulation
Prolonged Ventilation due to Ventilator Asynchrony
Diaphragm Muscle Injury due to Eccentric Contraction of the diaphragm caused by stimulation during inhalation

The following side effects are defined as IMPROBABLE (extremely unlikely or will not occur within lifetime of device). **This means that these improbable side effects listed below have never been seen before with devices like this one or with the VentFree device specifically:**

Improbable Side Effect
Shortness of Breath due to arrythmias caused by stimulation energy reaching the heart
Heart Attack or Chest Pain due to placement of electrodes on the chest area
Serious Organ Damage due EKG not detecting cardiac arrest
Electrocution
Cardiac Arrest due to leakage current
Reduced Tidal Volume caused by stimulation during inhalation
Barotrauma due to increase of transpulmonary pressure caused by stimulation during inhalation

Because VentFree™ is investigational there may be risks with its use that are not known at this time. Additionally, the device may malfunction.

Risks to Unborn Babies

There also may be risks to an unborn baby. You should not participate in this study if you are pregnant, and you should not become pregnant while on this study. If at any time you think you may be pregnant it is important to let the study staff know.

[END]
Risks of Participating in the Study

External echo/ultrasound:

Risks include discomfort from gel or pressure from hand piece.

Bedside Pulmonary Function test:

- Dizziness during the tests.
- Feeling short of breath.
- Coughing.
- Asthma attack brought on by deep inhalation.

Risks associated with participation in the study include loss of confidentiality. All of your protected health information will be secured to the extent possible according to local law.

Participating in this study could harm you in unknown ways.

WHAT ARE THE POSSIBLE BENEFITS OF BEING IN THE STUDY?

Subjects enrolled in the abdominal FES group may benefit by being removed from the ventilator sooner. There is no guarantee that you will have any benefit from the FES.

All subjects participating in the study may benefit from additional study assessments which have the intent to benefit you. The assessments are in addition to the standard of care you will still receive. They include:

- Specific Weaning Protocol: You will undergo a structured weaning (gradual reduction of ventilation) protocol. Previous research has shown that undergoing weaning protocols significantly reduces the duration of ventilation, weaning duration, and length of stay in the intensive care unit (ICU).
- 90-Day Follow-up Assessment: At the 90-day follow-up visit, you will receive additional assessments, which may aid in the early identification of any complications that may arise. This prompt detection would enable timely referrals or appropriate treatments, if necessary. During this visit, you will also be asked to complete a Quality of Life survey. If your survey score indicates a lower quality of life, your research team may recommend follow-up with your personal physician to ensure that further medical assessments are conducted.

The results of this research may further medical knowledge and improve future treatment of patients who need mechanical ventilation.

WHAT OTHER CHOICES DO I HAVE IF I DECIDE NOT TO BE IN THE STUDY?

You do not have to take part in this research study to receive treatment at this hospital. Instead of being on this study, you can complete the usual rehabilitation meant to reduce your dependence on the mechanical ventilator. You may be able to ask your doctor to use a specific weaning protocol and do a 90 day follow up assessment as part of your treatment. Your readiness to be weaned may occur using spontaneous breathing trials or using threshold inspiratory training.

Your study doctor will discuss options with you before you decide whether or not to participate in this study.

WILL IT COST ME ANYTHING TO BE IN THIS STUDY?

There is no cost to you for being in this study. The VentFree™ treatments and bedside pulmonary function tests will be provided at no cost to you or your health insurance. The charges for these items will be paid for by the sponsor.

You or your insurance will be responsible for the costs associated with your hospital stay and all routine care you receive to treat your condition. You may talk to the study staff and your insurance company about what is covered.

WHAT HAPPENS IF I AM INJURED OR HAVE COMPLICATIONS AS A RESULT OF THIS STUDY?

Every effort to prevent injury as a result of your participation will be taken. However, it is possible that you could develop complications or injuries as a result of participating in this research study. In the event of injury resulting from this research, medical treatment is available at this hospital. You should contact the study doctor as soon as possible so that prompt treatment can be provided.

Costs for the treatment of research-related injuries will be charged to your insurance company. Costs may be paid by the sponsor if for some reason the insurance company will not pay. If the sponsor does not pay, you will be billed for the costs. You will be billed for any deductible, co-insurance, and/or co-pay.

You will not lose any legal rights to pursue a claim through the legal system by signing this form.

WILL I BE PAID FOR BEING IN THIS STUDY?

You will be given $[Amount] after completing the final study visit to reimburse you for reasonable travel expenses and for your time in completing the visit and the questionnaire.

CAN I REFUSE TO PARTICIPATE OR STOP MY PARTICIPATION ONCE I HAVE STARTED?

Taking part in this research study is voluntary. You do not have to participate. If you choose to take part, you have the right to stop at any time. If you decide not to participate or if you decide to stop taking part in the research at a later date, there will be no penalty or loss of benefits to which you are otherwise entitled.

If you decide to stop participating, you should tell the study doctor so that appropriate continuation of care may be arranged. Information about you will no longer be sent to the study sponsor, and you may not have as many tests performed. Information that has already been gathered before you leave the study may still be used and given to others as described in this form. You will receive the same quality of care even if you withdraw your participation in the study.

During the research you will be provided with any significant new information that may affect your willingness to continue participating in this research. You may be asked to sign a new consent form with this new information.

CAN MY PARTICIPATION BE STOPPED EVENT IF I DON'T ASK TO STOP?

The study doctor may take you out of the research study without your permission. This may be to ensure your safety because you did not follow the study instructions, or for some other reason. Also, the sponsor may end the research study early. If your participation in the research ends early, you may be asked to visit the study doctor for a final visit.

WILL MY INFORMATION BE KEPT CONFIDENTIAL?

Your participation in this study is confidential. All information that is collected about you during this study will be kept confidential according to applicable law.

Your private information and your medical records including physician interactions/office visits, tests, interviews, questionnaires will be shared with individuals and organizations that conduct or watch over this research, including:

- The sponsor of this research study
- People who work with the sponsor
- Government agencies throughout the world, such as the Food and Drug Administration
- Representatives of the DoD
- The Institutional Review Board (IRB) that reviews this research

The FDA or other U.S. government agencies including the DoD, government agencies in other countries (including regulatory agencies), the IRB, and the sponsor may inspect and copy your medical records or other information about you related to this study. Some of these records could contain information that personally identifies you. Reasonable efforts will be made to keep the

personal information in your research record private and confidential but absolute confidentiality cannot be guaranteed.

Your health information will be processed for the purposes of this study both by computer and manually, during and after the study by the sponsor, its representatives (those who work for the sponsor to provide services related to the device and/or this study), the IRB, the hospital performing the study, and the study doctors and other healthcare personnel involved in the study. Your research records that are reviewed, stored, and analyzed at this hospital will be kept in a secured area and/or electronically on a secure server and backed up routinely. All records and reports required by or prepared in connection with this study shall be maintained by the hospital and the study doctor. The electronic data and results stored for this study by the sponsor will be kept in a database but will not contain details of your name or address.

Members of the Sponsor's team may be present during your stimulation procedures to provide support of the VentFree™ device.

The information collected about you may be used in several ways. The sponsor and its designees may use the information in any of the following ways:

- To analyze and make conclusions about the results of the study;
- In documents shared with government agencies and regulatory authorities throughout the world, including the FDA;
- For reporting adverse events to the FDA and other government health agencies;
- To provide overall study results to other study doctors, including in publications;
- To conduct new medical research, to re-examine the study results in the future or to combine your information with information from other studies; and
- To develop new medical products and procedures, and other product-development related activities in the U.S. and other countries.

You may withdraw or take away your permission to use and disclose your health information at any time. You do this by sending written notice to the study doctor. If you withdraw your permission, you will not be able to stay in this study. When you withdraw your permission, no new health information identifying you will be gathered after that date. Information that has already been gathered may still be used and given to others.

Your permission to use and share your identifiable information does not expire.

The results of this research may be published. However, your name and other identifying information will be kept confidential.

Your personal doctor may be informed of your participation in this research study, but only if you approve of this.

A description of this clinical trial will be available on http://www.ClinicalTrials.gov, as required by U.S. Law. This Web site will not include information that can identify you. At most, the Web site will include a summary of the results. You can search this Web site at any time.

Data or specimens collected in this research might be deidentified and used for future research or distributed to another investigator for future research without your consent.

WHO DO I CONTACT IF I HAVE QUESTIONS OR IF I AM INJURED?

You have the right to ask any questions you may have about this research. If you have questions, complaints, concerns, or believe you may have developed an injury related to this research, contact the Study Doctor at the number(s) listed in this document.

The IRB is a group of people who perform independent review of research as required by laws governing this type of research. For questions regarding your rights as a research participant, or other questions, concerns or complaints about the study, please contact WCG IRB at 855-818-2289 or clientcare@wcgclinical.com.

CONSENT TO BE IN THE RESEARCH

Before making the decision regarding enrollment in this research, you should have:

- Discussed this study with the study doctor for a member of the study team
- Reviewed the information in this form
- Had the opportunity to ask any questions you may have

Your signature below means that you have received this information, have asked the questions you currently have about the research, and have received answers to those questions. You will receive a copy of the signed and dated form to keep for future reference.

By signing this form, you acknowledge that:

- You voluntary agree to participate in this research study and will follow the study doctor’s instructions.
- You may contact the study doctor for further information regarding compensation for any study related injury.
- Refusal to participate or withdrawing your participation at a later date will not result in any penalty or loss of benefits to which you are otherwise entitled.
- If you are withdrawn from the study you will continue to receive standard of care treatment for your condition.
- You have read and understand this entire consent form and the study doctor or study staff has explained this study to me and reviewed this consent form with me.
- You have had ample time to ask questions and consider your participation in this research.

- You agree to the use of your personal health information for the purpose of this research and to have personal data transported outside the United States.
- Regulatory authorities, IRBs, and the sponsor involved in this research will have direct access to your medical records.
- I will receive a signed and dated copy of this consent form.

All subjects unable to consent are required to assent, unless the investigator determines that the capability of the subject is so limited that the subject cannot reasonably be consulted

If assent is obtained, have the person obtaining assent document assent on the consent form.

Participant:
By signing this consent form, you indicate that you are voluntarily choosing to take part in this research.

Signature of Participant	Date	Time	Printed Name

Participant's Legally Authorized Representative *(if applicable)*:
By signing below, you indicate that you give permission for the participant to take part in this research.

Print Name of Participant

Signature of Participant's Legally Authorized Representative	Date	Time	Printed Name

The signature of the participant's legally authorized representative is required for people unable to give consent for themselves.

Person Obtaining Consent:
Your signature below means that you have explained the research to the participant or participant representative and have answered any questions about the research.

Signature of Person Conducting Consent Discussion	Date	Time	Printed Name

Person Obtaining Assent

- ❑ I have explained the study to the extent compatible with the subject's capability, and the subject has agreed to be in the study.

OR

- ❑ The subject is not able to assent because the capability of the subject is so limited that the subject cannot reasonably be consulted.

_____________________________ ------------ --------- --------------------------
Signature of person obtaining assent Date Time Printed Name

Witness *(if applicable)*:

____________________________ ___________ _________ __________________________
Signature of Witness Date Time Printed Name

A witness is required when the participant cannot read or write. The consent form shall be read aloud and explained to the prospective participant, and the witness shall sign and personally date this form attesting that the information was accurately explained, and that informed consent was freely given.

For Participants who Gain Capacity Consent
Previously, you could not legally agree to take part in research. You took part in research based on the permission of someone else. Now that you can consent for yourself, you are being asked for your consent to continue to take part. Please read the entire document before signing below.

Your signature documents your consent to take part in this research.

____ ______________
Signature of adult subject capable of consent Date

____ ______________
Signature of person obtaining consent Date

Protocol # LM-VF-P3
IRB Approved Template
MUST BE APPROVED
FOR SITES BEFORE USE
AS MODIFIED
Oct 22, 2024

****For Sites in California****

AUTHORIZATION TO USE AND DISCLOSE INFORMATION FOR RESEARCH PURPOSES

What information may be used and given to others?

The study doctor will get your personal and medical information. For example:

- Past and present medical records
- Research records
- Records about phone calls made as part of this research
- Records about your study visits.

Who may use and give out information about you?

The study doctor and the study staff. They may also share the research information with an agent for the study doctor, if applicable.

Who might get this information?

The sponsor of this research. “Sponsor” means any persons or companies that are:

- working for or with the sponsor, or
- owned by the sponsor.

Your information may be given to:

- The U.S. Food and Drug Administration (FDA),
- Representatives from the DoD
- Department of Health and Human Services (DHHS) agencies,
- Governmental agencies in other countries,
- The institution where the research is being done,
- Governmental agencies to whom certain diseases (reportable diseases) must be reported, and
- Institutional Review Board (IRB).

Why will this information be used and/or given to others?

- to do the research,
- to study the results, and
- to make sure that the research was done right.

If the results of this study are made public, information that identifies you will not be used.

What if I decide not to give permission to use and give out my health information?
Then you will not be able to be in this research study.

May I review or copy my information?
Yes, but only after the research is over.

May I withdraw or revoke (cancel) my permission?
This permission will be good until December 31, 2070.

You may withdraw or take away your permission to use and disclose your health information at any time. You do this by sending written notice to the study doctor. If you withdraw your permission, you will not be able to stay in this study.

When you withdraw your permission, no new health information identifying you will be gathered after that date. Information that has already been gathered may still be used and given to others.

Is my health information protected after it has been given to others?
There is a risk that your information will be given to others without your permission.

Protocol # LM-VF-P3
IRB Approved Template
MUST BE APPROVED
FOR SITES BEFORE USE
AS MODIFIED
Oct 22, 2024

Authorization:
I have been given the information about the use and disclosure of my health information for this research study. My questions have been answered.

I authorize the use and disclosure of my health information to the parties listed in the authorization section of this consent for the purposes described above.

AUTHORIZATION SIGNATURE:

______________________________________ ____________
Signature of Subject/Legally Authorized Representative **Date**

____
Relationship to the Subject
