# Supplementary material for: Randomised, sham-controlled, double-blinded, multicentre international trial to evaluate the efficacy of the Ventfree Respiratory Muscle Stimulator to assist ventilator weaning in critically ill patients: a study protocol of a randomised controlled trial
Source: BMJ Open. 2026 Apr 21;16(4):e113540. doi: 10.1136/bmjopen-2025-113540 (PMC13110553; doi:10.1136/bmjopen-2025-113540)
Supplement: online supplemental file 2 [file bmjopen-16-4-s002.pdf]

APPENDIX WEANING PROTOCOL

**The following protocol includes criteria for weaning and extubation and is intended to standardize the weaning process across clinical trial sites. It is strongly recommended that each participating site follow this weaning protocol. The treating physician will make the final determination on readiness to wean and readiness to extubate based on clinical judgement. Reasons for deviating from this protocol will be documented in the applicable eCRF.**	
**READINESS TO WEAN**	The following readiness to wean criteria should be assessed at least once per day:<br>• Adequate oxygenation, as defined by SpO2 ≥ 90% on FiO2 ≤ 50% and PEEP ≤ 8 cmH_2O<br>• Adequate pulmonary function (RR ≤ 35/min)<br>• Stable cardiovascular status (systolic arterial blood pressure of 90-160 mmHg without or minimal vasopressors (≤0.2 µg/kg/min) and HR ≤ 140/min)<br>A spontaneous breathing trial (SBT) should be attempted when the readiness to wean criteria are met.<br>**NOTE: VentFree stimulation should not be performed during a SBT when the trial's purpose is to assess readiness for disconnection from IMV.**<br>**Example: If a participant's ventilator settings are being decreased (e.g., due to a neurological issue) without the intention of disconnection from IMV, VentFree stimulations CAN be continued.**
**SPONTANEOUS BREATHING (SBT) TRIALS**	SBT should be performed for at least 30 minutes and can occur by T-tube, low pressure support ventilation (PSV), or continuous positive airway pressure (CPAP). If PSV is used, the level of pressure support should be ≤ 8 cmH2O and PEEP should be ≤ 7 cmH_2O. If CPAP is used, the level of pressure should be set at 7 cmH_2O or less.<br><br>The SBT should be considered a failure if at least one of the following occurs:<br>• SpO_2 of < 90% with FiO_2 > 50 %<br>• Acute respiratory distress (RR ≥ 40/min, agitation, cyanosis)<br>• Hypertension, defined by systolic arterial blood pressure of ≥ 180 mmHg<br>• Hypotension, defined by systolic arterial blood pressure of ≤ 90 mmHg<br>• HR > 140, or increase of 20%<br>• Change in mental status
**CRITERIA FOR EXTUBATION**	A patient should be considered eligible for extubation if the following conditions are met:<br>• Completed a successful SBT (SBT did not meet any of the failure criteria listed above)

	• Cooperative cognitive state, as determined by the treating physician<br>• Adequate cough, as determined by the treating physician<br>• Frequency of suctioning is greater than every two hours
**PROPHYLACTIC INTERVENTION**	Prophylactic intervention, including non-invasive ventilation (NIV), high flow nasal cannula (HFNC), or both, should be considered for patients meeting any of the following criteria:<br>• ≥ 65 years of age<br>• Underlying cardiac disease<br>• Underlying respiratory disease
