# Supplementary material for: Randomised, sham-controlled, double-blinded, multicentre international trial to evaluate the efficacy of the Ventfree Respiratory Muscle Stimulator to assist ventilator weaning in critically ill patients: a study protocol of a randomised controlled trial
Source: BMJ Open. 2026 Apr 21;16(4):e113540. doi: 10.1136/bmjopen-2025-113540 (PMC13110553; doi:10.1136/bmjopen-2025-113540)
Supplement: online supplemental file 3 [file bmjopen-16-4-s003.pdf]

# APPENDIX BEDSIDE PFT PROTOCOL

| **Bedside pulmonary function tests (PFTs) The bedside PFTs should be conducted once the participant meets the awakening/comprehension criteria, but no sooner than 24 hours after being disconnected from the ventilator. The bedside PFTs are to be completed post initial disconnection from the ventilator only. Tests must be performed by experienced clinicians.** | |
|---|---|
| 1. Screen for awakening and comprehension | Observe responses to the following commands involving neck and face muscles:<br>• Open/close your eyes<br>• Look at me<br>• Open your mouth and put out your tongue<br>• Nod your head<br>• Raise your eyebrows after have counted up to five<br>Patients are considered “awakened” when they respond to all five of these orders. Bedside PFTs will be conducted for all enrolled participants. |
| 2. Conduct PFTs[1] | • Conduct on the first day of awakening.<br>• Patient should be in a semi-recumbent position at 45 degrees.<br>• Perform three (3) maneuvers and report all values on the applicable eCRF.<br>• Conduct cough peak flow (CPF), maximum expiratory pressure (MEP), and maximum inspiratory pressure (MIP) tests according to the methodology described below. |
| 3. Measure CPF[2] | • The bedside respiratory muscle strength system, Pneumotrac™ (Vitalograph; Lenexa, KS), will be used to conduct the CPF testing.<br>• Please ensure to use the supplied nose clip.<br>• Prior to conducting the CPF, the Pneumotrac software must be calibrated using the supplied 3L calibration syringe. This calibration is performed prior to the CPF ONLY.<br>• Upon initiation of testing, instruct the participant to breathe in deeply to allow for full inspiration to total lung capacity (TLC) then cough one time as hard as possible.<br>• Because this is an unfamiliar maneuver, careful instruction and encouraged motivation are essential.<br>• Patients often need coaching to prevent air leaks around the mouthpiece during the coughing efforts, and this may be helped by having them pinch their lips around the mouthpiece.<br>• Perform the CPF test only three (3) times. |
| 4. Measure MEP[3] | • The bedside respiratory muscle strength system, Pneumotrac™, will be used to conduct the MEP testing.<br>• Please ensure to use the supplied nose clip.<br>• Upon initiation of the testing, instruct the participant to breathe in deeply to allow for full inspiration to total lung capacity (TLC) then perform a maximum forceful expiration.<br>• The expiratory pressure must be maintained, ideally for at least 1.5 seconds, so that the maximum pressure sustained for 1 second can be recorded. The peak pressure may be higher than the 1 second of sustained pressure but is believed to be less reproducible. |

| | |
|---|---|
| | • Because this is an unfamiliar maneuver, careful instruction and encouraged motivation are essential.<br>• Patients often need coaching to prevent air leaks around the mouthpiece and to support the cheeks during the expiratory efforts, and this may be helped by having them pinch their lips around the mouthpiece.<br>• Perform the MEP test only three (3) times. |
| 5. Measure MIP[4] | • The bedside respiratory muscle strength system, Pneumotrac™, will be used to conduct the MIP testing.<br>• Please ensure to use the supplied nose clip.<br>• Upon initiation of the testing, instruct the participant to perform a maximum forceful inspiration after an expiration to residual volume (RV).<br>• The inspiratory pressure must be maintained, ideally for at least 1.5 seconds, so that the maximum pressure sustained for 1 second can be recorded. The peak pressure may be higher than the 1 second of sustained pressure but is believed to be less reproducible.<br>• Because this is an unfamiliar maneuver, careful instruction and encouraged motivation are essential.<br>• Patients often need coaching to prevent air leaks around the mouthpiece during the inspiratory efforts, and this may be helped by having them pinch their lips around the mouthpiece.<br>• Perform the MIP test only three (3) times. |

[1] Gibson, G. J. *et al.* ATS/ERS Statement on respiratory muscle testing. *Am. J. Respir. Crit. Care Med.* **166**, 518–624 (2002).
[2]Smina, M. *et al.* Cough peak flows and extubation outcomes. *Chest* **124**, 262-268 (2003).
[3]Medrinal, C. *et al.* Reliability of respiratory pressure measurements in ventilated and non-ventilated patients in ICU: an observational study. *Ann. Intensive Care* **8**, 1-5 (2018).
[4]Almeida CM, Lopes AJ, Guimarães FS. Cough peak flow to predict the extubation outcome: Comparison between three cough stimulation methods. *Can J Respir Ther.* **56**, 58-64 (2020).
