# Supplementary material for: Randomised, sham-controlled, double-blinded, multicentre international trial to evaluate the efficacy of the Ventfree Respiratory Muscle Stimulator to assist ventilator weaning in critically ill patients: a study protocol of a randomised controlled trial
Source: BMJ Open. 2026 Apr 21;16(4):e113540. doi: 10.1136/bmjopen-2025-113540 (PMC13110553; doi:10.1136/bmjopen-2025-113540)
Supplement: Supplementary file 4 [file bmjopen-2025-113540.reviewer_comments.pdf]

PEER REVIEW HISTORY

BMJ Open publishes all reviews undertaken for accepted manuscripts. Reviewers are asked to complete a checklist review form and are provided with free text boxes to elaborate on their assessment. These free text comments are reproduced below.

ARTICLE DETAILS

Title (Provisional)

A Randomised, Sham Controlled, Double-blinded, Multi-centre International Trial to Evaluate the Efficacy of the VentFree® Respiratory Muscle Stimulator to Assist Ventilator Weaning in Critically Ill Patients: A study protocol of a randomised controlled trial

Authors

McCaughey, Euan J; Mclachlan, Angus J; Cai, Jason; Cohen Freue, Guillermo; Demoule, Alexandre; Gotur, Deepa B; Hill, Nicholas S; Dimatteo, Christopher; Pena, Senen; Patel, Mayur; Girard, Timothy D; Heunks, Leo

---

VERSION 1 - REVIEW

**Reviewer**	**1**
**Name**	**Ridley, Emma**
**Affiliation**	**Monash University, Intensive Care Society Research Centre**
**Date**	**07-Feb-2026**
**COI**	**None**

Thank you for the opportunity to review this protocol that describes a sham controlled double blind RCT of a respiratory muscle stimulation device compared to sham control.

Overall the paper is very well detailed and I limited comments. The comments I do have aim to give certain aspects more clarity:

- Plans for consumer engagement: I note that the authors have selected that there has been no public engagement to this point. Generally inclusion of patients and/or families/carers is considered essential in the current research environment. While this may not have occurred to date- are there any plans to rectify this during the course of the trial or to aid dissemination of findings?

- An exclusion of>= 22 years of age is unusual in my setting- can this be described? Is it due to local jurisdiction or product age limits?

- Sham group amplitude: how will this be determined at eligibility screening? What does this mean exactly?

-Monitoring of discomfort: How will this be monitored? Will this impact blinding? Who will monitor this?

- Blinding: It is noted that patients are blinded and will be given instructions not to discuss their experience- these patients are MV and sedated? The are likely to not recall any of the procedures (even if awake and MV). Will you be confirming their memory or experience once extubated? What about the presence of family members at the bedside?

**Reviewer**	**2**
**Name**	**Bureau, Côme**
**Affiliation**	**CHU de Lille, Service de Médecine Intensive-Réanimation**
**Date**	**17-Feb-2026**
**COI**	**None**

This manuscript presents the protocol for a multicenter, randomized, sham-controlled, double-blind trial evaluating functional abdominal electrical stimulation (VentFree®) to facilitate weaning from mechanical ventilation in adult patients in intensive care. The protocol clearly describes an ongoing study, with recruitment dates, trial registration, and detailed methodology. The protocol is well structured, clinically relevant, and sufficiently detailed. The research question is important, and the pathophysiological rationale is consistent. As suggested by the BMJ recommendations for protocols, my comments are intended to potentially improve the clarity, transparency, and future interpretability of the results.

The manuscript explicitly states that recruitment is ongoing, with start dates and planned timelines; no results from the ongoing trial are presented, and pilot data are used solely as scientific justification. The inclusion criteria, intervention, endpoints, and statistical plan are well described.

Minor comments

The protocol clearly states that no independent data monitoring committee is planned because the study is considered low risk, which is understandable. Can you nevertheless specify who reviews the safety data during the study, if the adjudication of the endpoints is independent of the sponsor?

Is there a way to standardize the adjustment of stimulation intensity on a daily basis according to patient tolerance? (operator training, reproducibility, tutorial, measurement of treatment exposure)

Inclusion requires demonstrated abdominal contraction under stimulation. Is the proportion of patients excluded for this reason known?

It is stated that respiratory parameters are measured only after extubation when the criteria for awakening are met. What proportion of patients are expected not to meet these criteria? What is planned for these patients?

Will ultrasound training be required for evaluators?

VERSION 1 - AUTHOR RESPONSE

Reviewer: 1

Plans for consumer engagement: I note that the authors have selected that there has been no public engagement to this point. Generally inclusion of patients and/or families/carers is considered essential in the current research environment. While this may not have occurred to date- are there any plans to rectify this during the course of the trial or to aid dissemination of findings?

We appreciate the reviewer's comments regarding consumer engagement. This is not something that was initially incorporated into this study design when the project was conceived circa 2021. The reason for this was multifactorial. Firstly, it was somewhat difficult to identify who the appropriate consumer would be. As we are recruiting patients who are mechanically ventilated the majority of informed consent is taken from a legally authorised representative. As such, there was debate about whether ICU patients, or their family members, would be the most suitable consumer for this project. If the trial is successful, the decision to start this technology is likely to rest with the patients clinician. As such, we felt having a scientific advisory board comprised of leading ICU clinicians, who will ultimately use this technology, is a suitable alternative. Additionally, identifying appropriate consumers who could represent the opinions of ICU patients across all three continents of the study while taking into account local differences in healthcare funding and provision was challenging. As such, we were worried that including consumers from such different settings on our SAB would risk tokenism.

However, we agree with you that consumer engagement is an important aspect of any research project. As such, we will engage consumers in the dissemination of the results of this trial. The Lead PIs Girard and Heunks, who are world leaders in ICU research, will actively inform national and international patient organisations about the study results through conference discussions and workshops. The study sponsor, Liberate Medical, will also draft a lay summary of the trial that can be provided to study sites and will also be published on their website. This information has now been added from line 502.

An exclusion of>= 22 years of age is unusual in my setting- can this be described? Is it due to local jurisdiction or product age limits?

American device regulations define the paediatric population as any patient less than 22 years of age (see https://www.fda.gov/medical-devices/products-and-medical-procedures/pediatric-medical-devices). As such patients under 22 years of age were excluded from this trial. This information has now been added to the inclusion and exclusion criteria.

Sham group amplitude: how will this be determined at eligibility screening? What does this mean exactly?

The sham group intensity will also be determined during the screening abdominal FES session prior to randomization. This will be conducted by administering the abdominal FES at a frequency of 30 Hz, a pulse width of 350 μs, and an amplitude of stimulation insufficient to elicit abdominal wall muscle contraction. The initial stimulation amplitude will be set at 10 mA. In the event that ultrasound confirmation indicates muscle contraction at 10 mA, the amplitude will be systematically reduced in increments of 2 mA, with confirmation sought at each step until muscle contraction is no longer observed. This information has now been added to the manuscript.

It should be noted that although the participants allocated to the sham group will not receive stimulation at intensities great enough to cause muscle contraction, the screening abdominal FES test will be performed for all potential participants to ensure that the underlying muscle physiology of both groups is comparable.

Monitoring of discomfort: How will this be monitored? Will this impact blinding? Who will monitor this?

Discomfort during the intervention period will be monitored by the therapist applying the intervention using either the BPS or CPOT scales. This therapist will be unblinded to the group allocation and as such the monitoring of discomfort should not effect blinding of study staff or the integrity of the trial results.

Blinding: It is noted that patients are blinded and will be given instructions not to discuss their experience- these patients are MV and sedated? The are likely to not recall any of the procedures (even if awake and MV). Will you be confirming their memory or experience once extubated? What about the presence of family members at the bedside?

Participants will continue to receive Abdominal FES until they are discharged from the ICU (up to a maximum of 28 days). As such, they are unlikely to be reliant on MV and be sedated during the whole intervention period. As such, patients and their LAR will be given information not to discuss their perceptions of the stimulation. This information

will be repeated at a number of points during the study. This information has been enhanced in the manuscript.

Reviewer: 2

The protocol clearly states that no independent data monitoring committee is planned because the study is considered low risk, which is understandable. Can you nevertheless specify who reviews the safety data during the study, if the adjudication of the endpoints is independent of the sponsor?

An independent physician will serve as the study medical monitor and review all reportable events to monitor for incidence of serious events that would warrant modification or termination of the trial.

The medical monitor may recommend study modification or termination because of concerns over participant safety or issues. This notification will be submitted in writing to the Sponsor for consideration and final decision. This information has been added to the manuscript from line 453.

Is there a way to standardize the adjustment of stimulation intensity on a daily basis according to patient tolerance? (operator training, reproducibility, tutorial, measurement of treatment exposure)

All therapists delivering the intervention will be trained by the sponsor as to how to properly adjust the stimulation intensity. However, the need to adjust the intensity is multifactorial based on technical, physiological and patient changes. From a technical standpoint the electrode placement may vary slightly. This can change the electrodes location in relation to the motor points of the muscles, meaning the contraction intensity will vary. From a physiological point of view the patient may have variable amounts of abdominal oedema which will change the stimulation amplitude required to penetrate to the abdominal muscle motor points. Finally, patient tolerance of the stimulation (i.e. their acceptable level of discomfort) may vary on a daily basis. As such, we would not advocate standardisation of adjustment of stimulation intensity.

Inclusion requires demonstrated abdominal contraction under stimulation. Is the proportion of patients excluded for this reason known?

Based on data collected from the two pilot studies approximately 5% (5 out of 99) of patients screened for the study were excluded due to a lack of contraction of the abdominal muscles.

It is stated that respiratory parameters are measured only after extubation when the criteria for awakening are met. What proportion of patients are expected not to meet these criteria? What is planned for these patients?

The number of participants who will not meet the awakening criteria before ICU discharge is difficult to estimate. However, based on our pilot study performed in Australia, 4 of the 13 participants liberated from mechanical ventilation were unable to perform respiratory function measures. This was because 1 was delirious, 1 was transferred to another site and 2 had tracheostomies. The equipment for this trial has been updated to enable those with a tracheostomy to take part. As such, we believe the number of participants with incomplete respiratory function data will be minimal. It is also important to note that as respiratory function is a secondary outcome of the trial these participants will still be included in the final analysis despite the missing respiratory data.

Will ultrasound training be required for evaluators?

Training will be provided by the sponsor for all trial procedures (now added to Line 230 of the manuscript). This will include ultrasound training for those performing the eligibility screening. However, it should be noted that bedside ultrasound is now commonplace in ICUs and many of the study staff will already be familiar with using this technology.

VERSION 2 - REVIEW

Reviewer 1

Name Ridley, Emma

Affiliation Monash University, Intensive Care Society Research Centre

Date 19-Mar-2026

COI

Thank you, my comments have been addressed

Reviewer 2

Name Bureau, Côme

Affiliation CHU de Lille, Service de Médecine Intensive-Réanimation

Date 11-Mar-2026

COI

All my comments have been taken into account, I have no further remarks.
